# Differential ROS-Mediated Phosphorylation of Drp1 in Mitochondrial Fragmentation Induced by Distinct Cell Death Conditions in Cerebellar Granule Neurons

**DOI:** 10.1155/2021/8832863

**Published:** 2021-04-13

**Authors:** Carolina Cid-Castro, Julio Morán

**Affiliations:** División de Neurociencias, Instituto de Fisiología Celular, Universidad Nacional Autónoma de México, Apartado Postal, 70-253 Ciudad de México, Mexico

## Abstract

Reactive oxygen species (ROS) production has been associated with neuronal death. ROS are also involved in mitochondrial fission, which is mediated by Dynamin-related protein 1 (Drp1). The regulation of mitochondrial fragmentation mediated by Drp1 and its relationship to mitochondrial ROS (mtROS) in neuronal death have not been completely clarified. The aim of this study is to evaluate the role of mtROS in cell death and their involvement in the activation of Drp1 and mitochondrial fission in a model of cell death of cultured cerebellar granule neurons (CGN). Neuronal death of CGN induced by potassium deprivation (K5) and staurosporine (ST) triggers mitochondrial ROS production and mitochondrial fragmentation. K5 condition evoked an increase of Drp1 phosphorylation at Ser616, but ST treatment led to a decrease of Drp1 phosphorylation. Moreover, the death of CGN induced by both K5 and ST was markedly reduced in the presence of MitoTEMPO; however, mitochondrial morphology was not recovered. Here, we show that the mitochondria are the initial source of ROS involved in the neuronal death of CGN and that mitochondrial fragmentation is a common event in cell death; however, this process is not mediated by Drp1 phosphorylation at Ser616.

## 1. Introduction

Neuronal apoptotic death can be identified by multiple biochemical features [1–3] that involves the activation of several signaling pathways [3–6]. In addition to the classical biochemical changes, an elevation of ROS levels responsible for cell death is frequently reported [7–11]. The main sources of ROS implied in cell death are the mitochondria and the NADPH oxidases (NOX). In the first case, the elevation of ROS levels is caused by an impairment of the mitochondrial function and is mainly produced by complex I [12–15]. Depending on the cell death conditions, ROS are produced by the activation of different NOX homologues [5, 16, 17].

Additionally, the high levels of ROS observed during neuronal death have been associated with morphological changes of mitochondria [18, 19]. These alterations have been linked to a process known as mitochondrial dynamics that refers to a highly coordinated event responsible for the fusion and fission of the mitochondria [20–23]. This process is orchestrated by a family of GTPases called mitofusin 1 (Mfn1), mitofusin 2 (Mfn2), and optic atrophy 1 (Opa1) that are responsible for the fusion of the inner mitochondrial membranes. Other proteins, including Dynamin-related protein 1 (Drp1), are in charge of the scission of the outer and inner membranes [24–28]. An impairment in the expression or function of these proteins has been associated with pathologies of the nervous system such as Parkinson's disease, autosomal dominant optic atrophy, Charcot-Marie-Tooth disease, Leigh syndrome, and amyotrophic lateral sclerosis, among others [29–33].

Drp1 activation has been related to excessive mitochondrial fragmentation during neuronal death [19, 34–36]. The process involves the phosphorylation of Ser616, which generates the translocation of Drp1 from the cytoplasm to the outer mitochondrial membrane resulting in the initiation of the shortening of the mitochondria. Mitochondrial fission is usually related to mitochondrial dysfunction and increased production of mitochondrial ROS [37–43]. Since mitochondria are one of the main sources of ROS and the pivot organelle of apoptotic death, the regulation of its fragmentation mediated by Drp1 and its relationship to mitochondrial ROS have been implicated to apoptotic neuronal death, but their association is not still completely elucidated.

In previous studies, it has been shown that cerebellar granule neurons (CGN) must be cultured under depolarizing conditions to survive, which can be attained by maintaining neurons in high potassium (25 mM, K25). Under these conditions, treatment of CGN with staurosporine (ST) or potassium deprivation (K5) induces an early NOX-mediated production of ROS, activation of JNK and p38 signaling pathways, and apoptotic death [3, 8, 11], but no information is available on the role of mitochondrial ROS in the cell death and their involvement in the activation of Drp1 and mitochondrial fission. In the present study, we, therefore, assessed the effect of two cell death conditions, K5 and ST, on the mitochondrial (mtROS) and cytoplasmic ROS (ctROS) production, as well as their participation in the Drp1 activation and mitochondrial morphology.

Here, we found that K5 and ST induced an early increase in mtROS and a decrease in mitochondrial length, as well as a rise of Drp1 phosphorylation at Ser616 for K5, but a reduction for ST. A mitochondrial antioxidant inhibited cell death and the phosphorylation of Drp1 induced by K5, suggesting that mtROS play a role in CGN death. Although mitochondrial fragmentation is a common process in neuronal death of CGN, Drp1 phosphorylation at Ser616 seems not to be involved in this process. These findings place mtROS as key regulators of neuronal death in a manner independent of mitochondrial fragmentation.

## 2. Materials and Methods

Fetal calf serum, penicillin/streptomycin, and basal Eagle's medium were purchased from GIBCO, Invitrogen (Carlsbad, CA, USA). Dihydroethidium (DHE), MitoTracker green, and MitoTracker red CMH_2_XRos were purchased from Molecular Probes, Invitrogen (Carlsbad, CA, USA). Poly-l-lysine, trypsin, trypsin inhibitor, DNAse, cytosine arabinoside, DMSO (dimethyl sulfoxide), staurosporine, MitoTEMPO, and reagents for polyacrylamide gel electrophoresis (PAGE) were acquired from Sigma (St. Louis, MO, USA). Protease inhibitor cocktail tablets (Complete) were purchased from Roche (Mannheim, Germany), and phosphatase inhibitor minitablets were obtained from Thermo Scientific (Rockford, USA). ProSieve Quad Color Protein Marker was purchased from Lonza (Rockland, Maine, USA). Polyvinylidene fluoride (PVDF) membranes and Immobilon Western HRP substrate were acquired from Millipore (Concord Road, Billerica, MA, USA). Antibodies against Drp1, Drp1 (Ser616), and GAPDH were from Cell Signaling Technology (Danvers, MA, USA); peroxidase-conjugated anti-mouse was purchased from Jackson ImmunoResearch (West Grove, PA, USA).

### 2.1. Cell Culture

All animals used for the experimentation described in the present study were treated by the accepted standards of animal care and with the procedures approved by the local Committee of Research and Ethics of the Instituto de Fisiología Celular, Universidad Nacional Autónoma de México (protocol number: JMA120-17). The protocol used followed the Guidelines for the Care and Use of Mammals in Neuroscience as well as guidelines released by the Mexican Institutes of Health Research and the National Institutes of Health guide for the care and use of laboratory animals. All efforts were made to minimize animal suffering and to reduce the number of animals used.

Cerebellar granule neuron (CGN) cultures were prepared as previously described [44]. Briefly, cell suspensions dissociated from 8-day-old Wistar rat cerebellum were plated at a density of 265 × 10^3^ cells/cm^2^ in plastic dishes coated previously with poly-l-lysine (5 *μ*g/mL). The culture medium contained basal Eagle's medium supplemented with 10% (*v*/*v*) heat-inactivated fetal calf serum, 2 mM glutamine, 25 mM KCl, 50 𝜇g/mL streptomycin, and 50 U/mL penicillin. The medium described previously is referred in the text as K25. Cytosine arabinoside (10 𝜇M) was added 24 h after seeding to prevent the proliferation of nonneuronal cells. The cultures were kept at 37°C in an atmosphere of CO_2_ (5%) and saturated air with water vapor (95%). Cultures were maintained 7 days in vitro (DIV) in the depolarizing medium (K25), and cell death was induced by two different protocols: (1) the neurons were transferred to a serum-free medium containing 5 mM KCl (referred as K5 or potassium deprivation) or (2) cultures were added with 0.5 𝜇M of ST.

### 2.2. Determination of Cytoplasmic ROS Levels

CGN were cultured in K25 medium during 7 DIV and then treated with K5 medium or ST as previously described. After the indicated times, the CGN were incubated with 3.2 𝜇M of DHE for 30 min at 37°C and cells were observed in an epifluorescence microscope with a rhodamine filter. Cells were photographed, and fluorescence intensity was measured with the ImageJ platform.

### 2.3. Determination of Mitochondrial ROS Levels

CGN were cultured in 35 mm Petri dishes during 7 DIV, and cells were preincubated for 30 min with MitoTracker red CMH_2_XRos (100 nM) at 37°C. Cells were then subjected to the cell death conditions for the indicated times, and pictures were collected by a LSM 710-Zeiss microscope at 740/599 nm excitation/emission, with a 63x immersion objective. Fluorescence intensity was measured with the Fiji ImageJ platform.

### 2.4. Mitochondrial Imaging

CGN were cultured on cover glass (FluoroDish™) in 35 mm Petri dishes, and after 7 DIV, the cells were treated with K5 medium or ST during the indicated times. Cells were then incubated with MitoTracker green (100 nM) for 30 min at 37°C, and cells were then washed twice with Locke medium (154 mM NaCl, 25 or 5 mM KCl, 3.6 mM NaHCO_3_, 2.3 mM CaCl_2_, 5.6 mM glucose, and 10 mM HEPES) and imaged with Eclipse-Ti-S Nikon by using a 63x oil objective with a fluorescein filter. The mitochondrial length was measured by using the Fiji ImageJ platform by selecting 20 individual mitochondria per image. After calibrating the images with the objective 60x of the Eclipse-Ti-S Nikon microscope, we draw a line over the individual mitochondria and we measured the mitochondrial length.

### 2.5. Western Blot

CGN were cultured in a K25 medium for 7 DIV and then switched to K5 medium or treated with ST at different times. Cells were washed twice in ice-cold PBS and were homogenized in lysis buffer (25 mM Trizma, 50 mM NaCl, 2% Igepal, 0.2% SDS and complete protease inhibitors, pH 7.4). Homogenates were centrifuged at 4,500 rpm for 5 min, and the supernatants were recovered. The protein concentration of homogenates was estimated by the Lowry method. Cell homogenates (30 𝜇g protein per lane) were subjected to 10% SDS-PAGE and transferred to PVDF membranes at 100V for 1.5 h. The membranes were blocked with Tris-buffered saline (TBS)/Tween 20 (TTBS) buffer (100mM Tris-HCl, 150 mM NaCl, and 0.1% Tween, pH 7.4) containing 5% or 2.5% nonfat dry milk at 4°C per one hour and were incubated overnight at 4°C with the primary antibodies. After washing, the blots were incubated with peroxidase-conjugated anti-mouse (1 : 10,000) or peroxidase-conjugated anti-rabbit (1 : 10,000) for 1 h at room temperature. Bands were visualized using chemiluminescence according to the manufacturer's recommendations and exposed to Kodak BioMax-Light Film.

### 2.6. Viability

We evaluated cell viability by the MTT (3-(4,5-dimethylthiazol-2-yl)-2,5-diphenyltetrazolium bromide) reduction technique, which is based on the ability of mitochondrial succinate dehydrogenase to transform MTT to formazan blue. The amount of formazan produced is directly proportional to the number of viable cells present in the culture. The cells were incubated with MTT (100𝜇M) for 15min at 37°C at the indicated times. Cells were then washed, and formazan blue crystals formed were dissolved with DMSO and measured in the spectrophotometer at 570nm.

### 2.7. Statistical Analysis

Data are presented as mean ± SE, and the statistical significance of the results was determined by one-way analysis of variance (ANOVA), followed by Fisher's test. *p* values less than 0.05 were considered statistically significant.

## 3. Results

### 3.1. K5 and ST Induce an Elevation of ROS Levels

**Figure 1:**
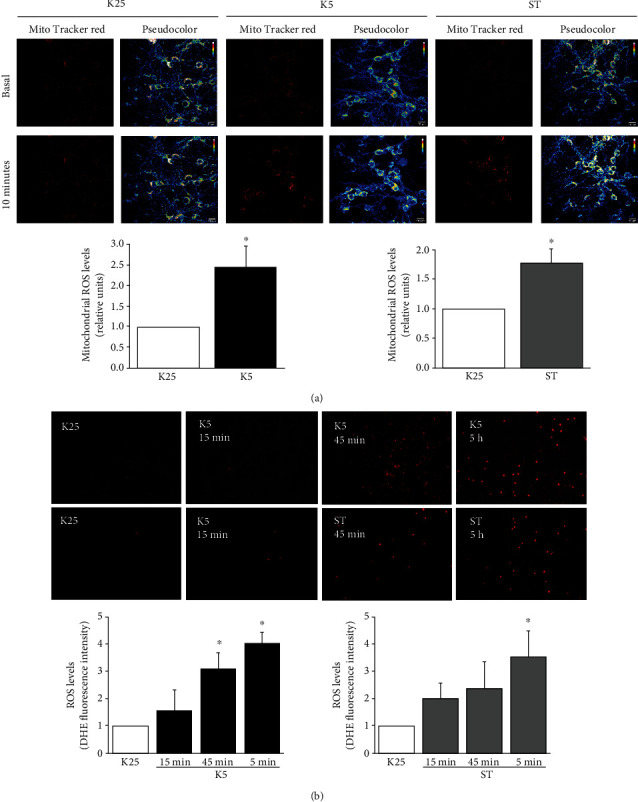
ROS levels induced by cell death conditions. The levels of mitochondrial and cytoplasmic ROS were measured at different times after potassium deprivation (K5) or staurosporine (ST) treatment. (a) CGN stained with MitoTracker red were imaged after 10 minutes to determine the mtROS levels under control (K25), K5, and ST treatments. The graphs show mitochondrial ROS production measured as indicated in Materials and Methods. (b) Cytoplasmic ROS were determined with DHE staining under control conditions (K25) or after 45 min and 5 h of K5 and ST treatment. The graphs show cytoplasmic ROS production measured as indicated in Materials and Methods. Bars are the means ± SE of three independent experiments. ^∗^*p* < 0.05 vs. K25.

In order to determine whether K5 or ST generated changes in ROS levels, we assessed a temporary course of mtROS and ctROS levels. First, we evaluated the effect of K5 and ST (0.5𝜇M) on mtROS and we observed a ROS increase after 10min in both death conditions. Remarkably, K5 condition induced more than a twofold elevation in the levels of ROS (Figure 1(a)), while ST showed a significant increase in mtROS by about 65% (Figure 1(a)).

Regarding ctROS levels, we found, in a temporal course measurement, that both K5 and ST induced a significant increase after 45 min and 5 h, but not at 15 min (Figure 1(b)). These data indicate that the mitochondria are the first source of ROS during the apoptotic process and a subsequent increase of ROS levels occurs in the cytosol, as previously demonstrated [45].

### 3.2. Elevation of ROS Correlates with Loss of Viability in Neurons Treated with K5 and ST

**Figure 2:**
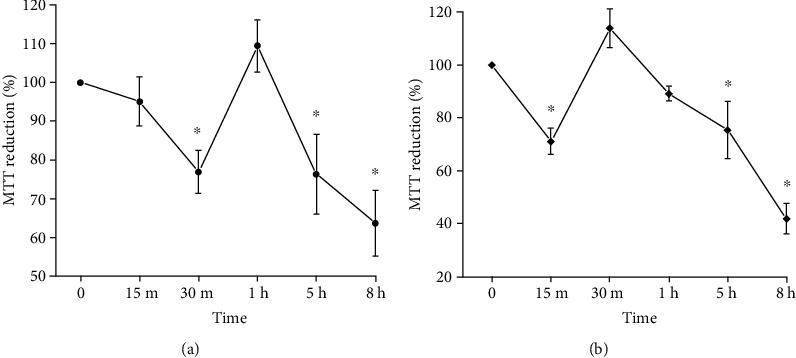
Time course of MTT reduction of CGN treated with K5 and ST. Cell viability was evaluated by MTT reduction after different times in cell death conditions. (a) Temporal course of the viability of CGN treated with K5 (●). (b) Viability of CGN treated with ST (♦). Symbols ● and ♦ show the mean ± SE of the percentage of viability compared with time 0 (K25) of three independent experiments. ^∗^*p* < 0.05 vs. time 0 (K25).

Because ROS occurs at different times of neuronal death in both models, it is important to know whether ROS elevation correlates with the loss of viability. For this, we evaluated the ability of neurons to reduce MTT as a viability indicator, over a period of 15 min to 8 h of K5 and ST treatment. Under these conditions, we observed a decrease in MTT reduction of 23% after 30 min that continues decreasing for 8 h of K5 treatment (Figure 2(a)). Similarly, in neurons treated with ST (Figure 2(b)), the reduction in MTT decreased by 28% after 15 min of treatment that continued decreasing after 8 h of treatment. These results suggest that the viability is compromised from the first minutes of the process of cell death induced by both stimuli and that the initial loss of viability correlates with the early mtROS production and with the progressive rise of ctROS.

### 3.3. mtROS Are Involved in the Neuronal Death Induced by K5 and ST

**Figure 3:**
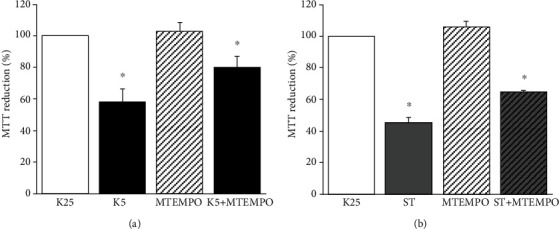
Role of mtROS in the viability of CGN treated with K5 and ST. Cell viability was evaluated by MTT reduction after 24 h of death induction in cells pretreated for 30 min with the mitochondrial antioxidant MitoTEMPO (10 *μ*M). (a) Viability of CGN treated with K5. (b) Viability of CGN treated with ST. Bars show the mean ± SE of the percentage of viability compared with the control (K25) of three independent experiments. ^∗^*p* < 0.05 vs. K25.

To evaluate the contribution of mtROS in cell death of CGN, cultures were treated with MitoTEMPO and we measured cell viability in cells treated with K5 or ST. After 24 h, K5 and ST treatment reduced the neuronal viability to 58% and 45.45%, respectively. When cells were pretreated with MitoTEMPO for 30 min, the observed decrease in cell viability was prevented to 80.11% (Figure 3(a)) and 64.87% (Figure 3(b)), respectively. These results suggest that mtROS production is a critical early signal in neuronal death.

### 3.4. Cell Death Conditions Induce Changes in Mitochondrial Morphology

**Figure 4:**
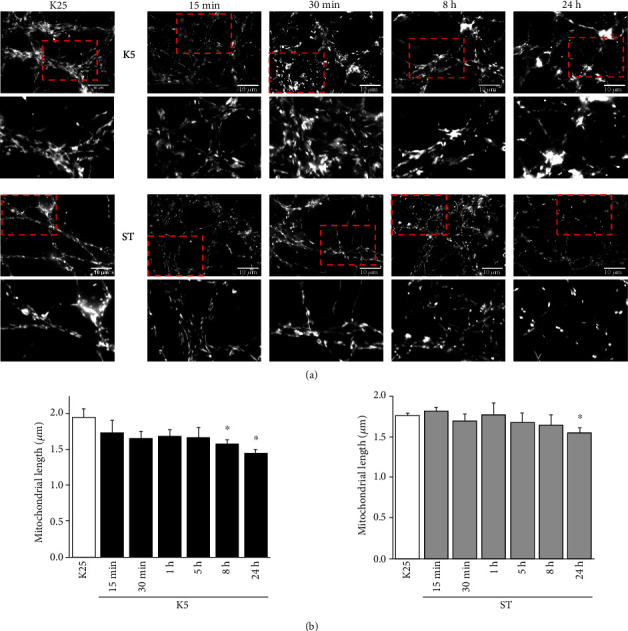
Morphological changes in mitochondria of CGN after cell death induction. Temporal course of CGN stained with MitoTracker green and treated with K5 and ST. (a) Image of mitochondrial morphology of CGN after potassium deprivation or ST at different times. The red arrows indicate the interconnected mitochondria in K25 condition and fragmented mitochondria after 8 and 24 h of treatment. (b) The graphs show the mitochondrial length in a temporal course during cell death. The bars represent the mean ± SE of three individual experiments. ^∗^*p* < 0.05 vs. K25.

In numerous models of cell death, mitochondrial morphological changes have been reported. These changes are characterized by swelling, rounding, and shortening of mitochondria that has been identified as mitochondrial fragmentation [46–48]. Under our conditions, we observed that CGN subjected to K5 showed mitochondrial morphological changes at 8 h (Figure 4(a)). These neurons showed rounded and shorter mitochondria when compared to those observed in control conditions (K25); the average length of mitochondria was reduced by 18% at 8 h and 25% after 24 h of K5 treatment (Figure 4(b)). In the case of ST treatment, rounded mitochondria were observed starting at 8 h and a significant mitochondrial shortening of 11.86% was observed after 24 h of treatment (Figure 4(b)). When we quantified the number of mitochondria, we did not observe any difference between the evaluated conditions (not shown). These results demonstrate that a decrease in mitochondrial length is a common event during neuronal death induced by different apoptotic stimuli.

### 3.5. K5 Induces mtROS-Dependent Drp1 Phosphorylation

**Figure 5:**
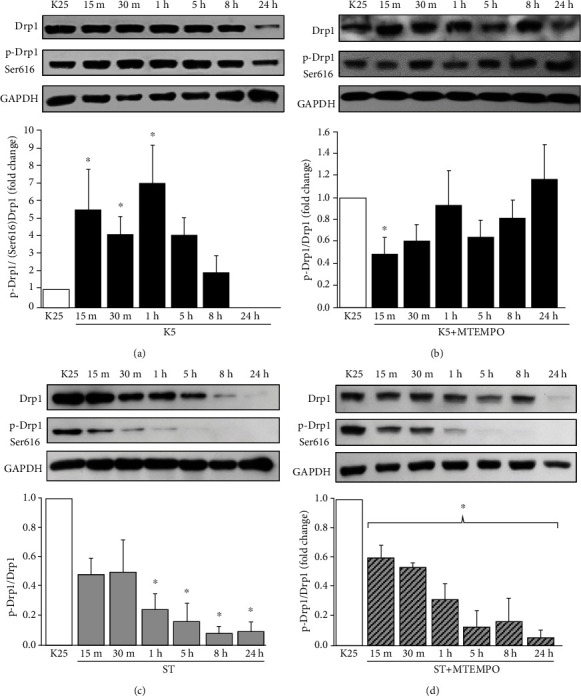
Effect of mtROS in the activation of Drp1 induced by K5 and ST. The levels of total Drp1 and Drp1 phosphorylated at Ser616 (p-Drp1) were evaluated in a temporal course in lysates of CGN pretreated for 30 min with the mitochondrial antioxidant MitoTEMPO under death conditions. The levels of the protein were determined by Western blot analysis as indicated in Materials and Methods. (a) Levels of p-Drp1 (Ser616) from CGN treated with K5. (b) Levels of p-Drp1 from CGN pretreated with MitoTEMPO and treated with K5. (c) Levels of p-Drp1 (Ser616) from CGN treated with ST. (d) Levels of p-Drp1 from CGN pretreated with MitoTEMPO and treated with ST. GAPDH was used as loading control. The bars show the densitometric ratio between p-Drp1 and Drp1 that were normalized to the control K25. Values are the mean ± SE of three individual experiments. ^∗^*p* < 0.05 vs. K25.

In order to clarify the role of potassium deprivation in the process of mitochondrial fission, we evaluated the activation of Drp1 measured as Drp1 phosphorylation at Ser616. We carried out a temporal course of K5 treatment, and we observed a rise in Drp1 phosphorylation after 15 min of stimulation, which remained constant for 8 h of treatment. After 24 h, Drp1 phosphorylation decreased (Figure 5(a)). As mentioned above, mtROS elevation was detected early during the cell death process (Figure 1(a)); thus, we evaluated the effect of the mitochondrial antioxidant MitoTEMPO on Drp1 phosphorylation in a temporal course. Data showed that MitoTEMPO inhibited the Drp1 phosphorylation induced by K5 from 15 min to 24 h of treatment (Figure 5(b)). These data suggest that mtROS are required for Drp1 activation, evidenced as Ser616 phosphorylation, during potassium deprivation.

### 3.6. ST Decreases Drp1 Phosphorylation Levels

Interestingly, when we explored the effect of ST on Drp1 phosphorylation in a time course assay, we found an early decrease in the phosphorylated form of Drp1 starting at 1 hour and further reducing after 5, 8, and 24 h (Figure 5(c)). The observed decrease in phosphorylation was not modified by treatment with MitoTEMPO (Figure 5(d)). These data suggest that mitochondrial fragmentation induced by ST treatment (Figure 5(c)) is not mediated by either Drp1 Ser616 phosphorylation, which is unrelated to mtROS production.

### 3.7. Mitochondrial Fission Induced by Cell Death Is Not Prevented by Treatment with a Mitochondrial Antioxidant

**Figure 6:**
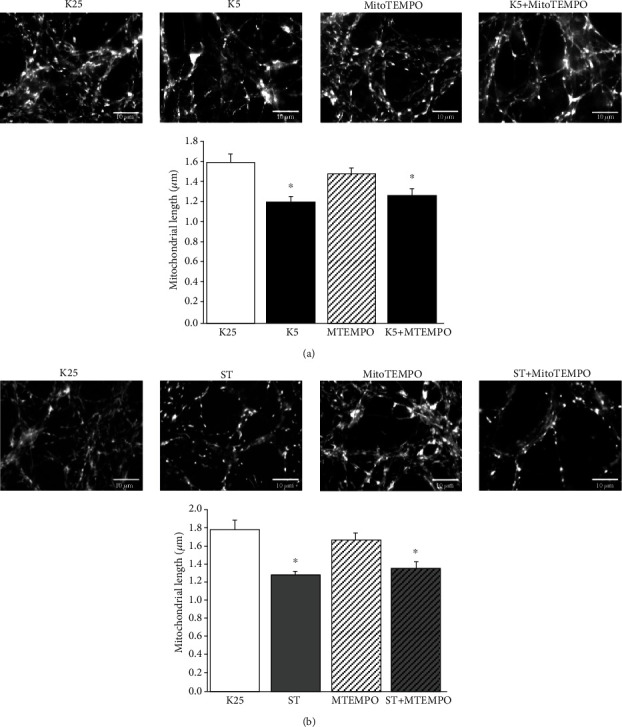
Role of mtROS in the morphological changes of mitochondria in CGN treated with cell death conditions. CGN stained with MitoTracker green and pretreated with MitoTEMPO (10 *μ*M) were stimulated with K5 or ST, and neurons were imaged after 24 h. (a) Image of mitochondrial morphology of CGN after potassium deprivation. (b) Image of mitochondrial morphology after ST treatment. The bars show the mitochondrial length in *μ*m measured after 24 h of treatment with the death conditions. The bars represent the mean ± SE of three individual experiments. ^∗^*p* < 0.05 vs. K25.

Since mtROS mediated the phosphorylation of Drp1 (Ser616) induced by potassium deprivation, we examined whether MitoTEMPO affected the observed effect of K5 on mitochondrial morphology; however, we did not observe any effect of MitoTEMPO on the decrease in mitochondrial length induced by K5 at 24 h (Figure 6(a)). Similar results were obtained for ST (Figure 6(b)). MitoTEMPO alone did not exert any effect on mitochondrial length (Figures 6(a) and 6(b)). Additionally, when cultures were incubated with 10 *μ*M MDiVi-1, an inhibitor of Drp1, the cell death of CGN induced by K5 or ST was not reduced (Suppl. Fig. 1).

## 4. Discussion

One of the major findings in this study was the observation of an early increase of mtROS production in response to two different cell death conditions: K5 and ST (Figure 1(a)). Interestingly, the observation that mitochondrial ROS scavenging ameliorated neuronal viability under K5 and ST treatments indicates that the observed increase in mtROS is an event that contributes to neuronal death. This proposal is supported by previous studies in other experimental models where antioxidants improved mitochondrial function [49, 50]. The protective effect of MitoTEMPO on cell viability in both models was partial, suggesting that other sources of ROS are involved in the cell death process.

Mitochondria is a hub in many physiological functions and one of the main sources of ROS in the cell. There is a large body of evidence showing the role of mitochondrial ROS (mtROS) in the regulation of many physiological processes [51]. For example, mtROS are involved in neuronal differentiation [52] and cell proliferation [53]. Some of the mtROS actions are mediated by the regulation of calcium transport into the cell and intracellular stores [54, 55]. Accordingly, deregulation of mtROS can lead to pathological conditions. It is known that the release of mtROS by mitochondrial permeability transition pore opening is a crucial step in the pathogenesis of diverse diseases [56–58]. Particularly, alterations in mtROS have been related to several neurodegenerative diseases [59]. For example, mtROS have been associated with an alteration of the long-term potentiation in an Alzheimer's disease (AD) model [60] and the use of mitochondrial antioxidants prevents the expression of the characteristics of AD in mice [61]. In addition, mitochondrial fission is related to an increased mtROS levels in an AD model [39]. Other neuropathologies related to mtROS overproduction includes frontotemporal dementia [62] and Parkinson's disease [63], among others. Thus, mtROS are essential to maintain physiological homeostasis of the cell, but a misbalance can cause serious pathological alterations.

In this regard, our group previously demonstrated, and we corroborated here (Figure 1(b)), that cytoplasmic ROS elevation is a determinant process in neuronal death [3, 6, 64]. The observed increase in ROS levels has been associated with the promotion of apoptosis, but the specific mechanism remains elusive. We and others have shown that NOX is a crucial ROS source implicated in apoptosis [8, 16, 65]. Here, we showed an early production of both mtROS and ctROS induced by K5 and ST. The initial ROS produced by mitochondria could be related to ctROS produced later by NOX. Previous studies have suggested an interrelationship between mtROS and NOX activation [65], and a feedback mechanism between mtROS and ROS generated by NOX has also been proposed [66].

We and others have demonstrated that an increase of ROS levels is related to the progression of cell death [8, 15, 45, 54]. Thus, we assessed the capacity of neurons to reduce MTT and we observed that K5 and ST induce a reduction in the viability from the first 15-30 min that continued decreasing for 8 h (Figure 2). These results suggest that the neuronal death process is an event triggered from the first minutes of the treatments as it occurs for the increase of ROS levels (Figures 1 and 2). Previously, the early impairment of viability has been reported in CGN under oxidant conditions [55], showing that ROS has a role in the sudden loss of viability and acts as a determinant to the neuronal fate. In our study, we confirmed that two death conditions induced mtROS and ctROS levels and this correlates with a rapid loss of viability.

There are evidence supporting the idea that altering mitochondrial function by K5 and ST is an early episode likely involved in the death of cerebellar granule neurons and that K5 and ST could have different actions in mitochondrial activity. For example, some studies suggest that apoptotic conditions alter the mitochondrial function early in the cell death process. For example, Jekabsons and Nicholls [67] showed a decrease in the oxygen consumption from the first few minutes of potassium deprivation.

A recent study has shown that the suppression of ROS production from both sources was not additive in preventing A*β* toxicity of cultured cortical neurons [68]. This result is in agreement with the observed partial protective effect of MitoTEMPO observed in our model (Figures 2(a) and 2(b)) and supports the idea that other intracellular signals besides mtROS contribute to the neuronal death.

Changes in mitochondrial morphology and their relationship to the process of neuronal death have gained relevance as an essential issue in the progression of neurodegeneration caused by harmful stimuli [39, 69, 70]. Interestingly, and consistent with previous studies, we observed morphological alterations in mitochondria at different times of treatment with both cell death conditions. CGN maintained in basal conditions showed highly connected mitochondria, which after several hours of treatment with K5 or ST became shorter and rounded along neurites (Figure 3), in agreement with previous studies [47, 50].

The impairment of mitochondrial morphology has been related to increased ROS levels in different experimental models [43, 46, 50, 71], including the use of hydrogen peroxide in neuroblastoma cells and cultured hippocampal neurons [50, 70]. Particularly, it has been reported that a reduction in mtROS decreased the mitochondrial fragmentation [50, 72]. Since in our study, the use of a mitochondrial antioxidant ameliorated the loss in cell viability of the neurons (Figure 2), we evaluated the role of mtROS on the mitochondrial morphology under cell death conditions; however, we found that MitoTEMPO did not prevent mitochondrial fragmentation in any of the cell death conditions studied (Figure 6). It remains to evaluate whether ctROS are involved in the morphological changes induced by K5 and ST.

The redox balance has been linked to the regulation of the core of the mitochondrial dynamics regulating proteins [73]. Drp1 is the main protein involved in the regulation of mitochondrial fission [26, 34, 74]. Mitochondrial fragmentation requires the translocation of Drp1 to the outer mitochondrial membrane [75], which involves its phosphorylation at several sites, including Ser616 and Ser637 [76, 77]. To further assess the role of mtROS in mitochondrial fission, we evaluated the activation of Drp1 mediated by its phosphorylation at Ser616 in neurons treated with K5 or ST. In the case of K5, we found an increase in Drp1 phosphorylation that correlated with the observed increase in mtROS and mitochondrial fission (Figure 4(a)), in agreement with previous studies [42, 71]. Moreover, we observed that MitoTEMPO significantly reduced the increase of p-Drp1 induced by K5 (Figure 4(b)), suggesting that Drp1 phosphorylation could be mediated by the mtROS induced by potassium deprivation.

In contrast to K5, although ST induced a rise in mtROS that correlated with decreased mitochondrial length, we did not observe any activation of Drp1 measured as phosphorylation at Ser616. In fact, we observed a marked decrease in both total Drp1 and p-Drp1 by ST (Figure 5(a)). In addition, MitoTEMPO did not modify the decrease in total Drp1 and p-Drp1 induced by ST (Figure 5(b)). Unexpectedly, we observed a decrease in Drp1 total levels from 15 min to 24 h in neurons treated with ST (Figures 5(c) and 5(d)). A decrease in phosphorylated Drp1 levels has been reported to promote mitochondrial elongation in the hippocampus [78]. Thus, these results show that Drp1 and mtROS do not mediate the mitochondrial fission induced by ST. It is possible that the observed Drp1 degradation by ST could be mediated by a mechanism dependent on the proteasome, as it has been observed in other models of neuronal death [79].

Alternatively, other Drp1 phosphorylation sites could be responsible for mitochondrial fission induced by ST, as it has also been suggested during neurodegeneration [80]. Although the most commonly reported phosphorylation of Drp1 is at Ser616 [29, 39, 81], a decrease in phosphorylation at Drp1 Ser637 has also been shown in hippocampal neurons [76, 82, 83]. In addition, the phosphorylation at Ser585 was related to an enhanced mitochondrial fission in CGN in response to excitotoxicity [84]. Thus, we cannot discard other possible sites of Drp1 phosphorylation involved in the process of mitochondrial fission.

A possible explanation for the observed differences in Drp1 phosphorylation by K5 and ST could be the distinct signaling pathways activated by each condition. Drp1-dependent mitochondrial fragmentation is regulated by several kinases, including CDK5 [84], CaMKII [82, 83], ERK1/2, PKC, JNK, and p38 in different models [71, 85, 86]. We have previously reported that both models of apoptotic death showed differences in their molecular mechanisms of action. K5 induces a reduction in cytoplasmic calcium, while ST induces an early increase of calcium [44, 87]; K5 evokes the release of K+, while ST produces a Cl- release [88], and although both conditions activate NOX, only ST induces the activation of NOX2 [16]. Finally, we have also highlighted the differential activation of signaling pathways by K5 and ST during apoptotic neuronal death; the effect of K5 was mediated by JNK, while ST required p38 activation [6]. We hypothesize that any of these differences could be responsible for the discrepancies in K5 and ST conditions in the Drp1 phosphorylation.

In the present study, we observed mitochondrial fragmentation in both experimental models. However, in the case of the neurons subjected to K5, the total abolition of the Drp1 phosphorylated levels by MitoTEMPO was not enough to reduce mitochondrial fission. In the ST model, we also observed a significant mitochondrial fragmentation even with very low levels of total and phosphorylated Drp1 at Ser616. It is worth mentioning that we do not observe any effect of the putative Drp1 inhibitor MDiVi-1 on the viability of CGN treated with K5 or ST suggesting that Drp1 could not be critical for cell death of CGN. It should be noted that in other models, including cell reprogramming [89] or cell proliferation in tumor growth [90], the mechanisms and consequences of mitochondrial fission might be different from those of neuronal death.

These results suggest alternative mechanisms to induce mitochondrial fission. It has been recently proposed that actin cytoskeleton modulates mitochondrial morphology changes [91, 92] that are involved in neuronal death [93], but the role of actin-cofilin has not been explored in neurons. Particularly, cofilin seems to participate in the mitochondrial fission and apoptosis through the dephosphorylation of Drp1 at Ser637 [94].

## 5. Conclusions

**Figure 7:**
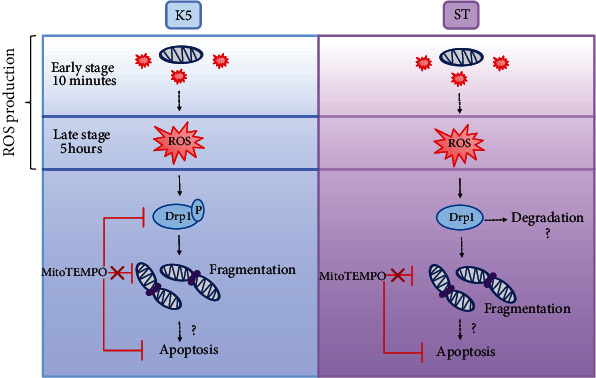
K5 and ST induce mtROS and mitochondrial fragmentation not mediated by Drp1 p-Ser616 during neuronal death. The model proposed for both apoptotic conditions comprises an early stage of ROS produced by mitochondria, which was shown in this study, as well as a late stage of ROS generated by NOX [16, 45]. Based on the finding that MitoTEMPO inhibited Drp1 phosphorylation, we propose that the mtROS are involved in the Ser616 phosphorylation of Drp1. Further, we observed mitochondrial fragmentation and finally neuronal death. However, the inhibition of Drp1 activation by MitoTEMPO did not reduce mitochondrial fragmentation; nevertheless, MitoTEMPO reduced neuronal death. This suggests that mitochondrial fragmentation mediated by Drp1 Ser616 is not involved in CGN apoptotic death. On the other hand, ST treatment (right panel) also induced an increase in both mitochondrial and cytoplasmic ROS levels. Here, we found that ST markedly reduced total Drp1 and Ser616 Drp1 phosphorylation levels and, as with K5, MitoTEMPO did not inhibit the mitochondrial fragmentation, but it prevented the neuronal death. As for K5, we observed that Drp1 phosphorylation at Ser616 is not related to either mitochondrial fragmentation or neuronal death induced by ST. Although Drp1 activation is a determinant of the mitochondrial fission in other models of death, this does not seem to occur in our model, at least through its phosphorylation at Ser616 Drp1, which does not play a role in mitochondrial fragmentation and cell death of CGN. Based on these findings, we propose that there exists an alternative mechanism that regulates the mitochondrial fragmentation in CGN.

In conclusion, our findings suggest that mtROS are necessary for the process of neuronal death, but not for the mitochondrial fragmentation. However, the cell death conditions induce mitochondrial fragmentation in CGN. In addition, mitochondrial fragmentation and neuronal death of CGN seem not to be mediated by Drp1 phosphorylation at Ser616. Our data suggest that mitochondrial fragmentation is carried out by different mechanisms depending on the cell death condition. The details of the suggested mechanism are described in Figure 7.

More experiments are needed to explore the relationship among mtROS, mitochondrial dynamics, and cell death induced by different conditions, as well as the fine mechanisms involved, including the alternative sites of Drp1 phosphorylation. Future experiments may clarify how the different sources of ROS, including NOX, may induce mitochondrial fragmentation and cell death. Furthermore, other core proteins of the mitochondrial dynamic process, like Opa1 and mitofusins, could have a specific role in K5 and ST induction of cell death.

## Data Availability

The data used to support the findings of this study are available from the corresponding author upon request.

## Supplementary Materials

Supplementary Materials Supplementary Figure 1 Effect of MDiVi-1 on cell viability of CGN treated with K5 and ST. Cells were cultured for 7 DIV and treated with staurosporine (0.5 *μ*M) (ST) or transferred to a 5 mM KCl-containing medium (K5) in the presence or absence of the Drp1 inhibitor MDiVi-1 (10 *μ*M); the MDiVi-1 was preincubated 30 min before the corresponding treatment. Cell viability was evaluated as MTT transformation after 24 h. Values are *means* ± *SE* of three independent experiments. ^∗^Significantly different from control (K25) (^∗^*p* < 0.05).
