# Supplementary figures and images for: Differential ROS-Mediated Phosphorylation of Drp1 in Mitochondrial Fragmentation Induced by Distinct Cell Death Conditions in Cerebellar Granule Neurons
Source: Oxid Med Cell Longev. 2021 Apr 13;2021:8832863. doi: 10.1155/2021/8832863 (PMC8060094; doi:10.1155/2021/8832863)

A) B)

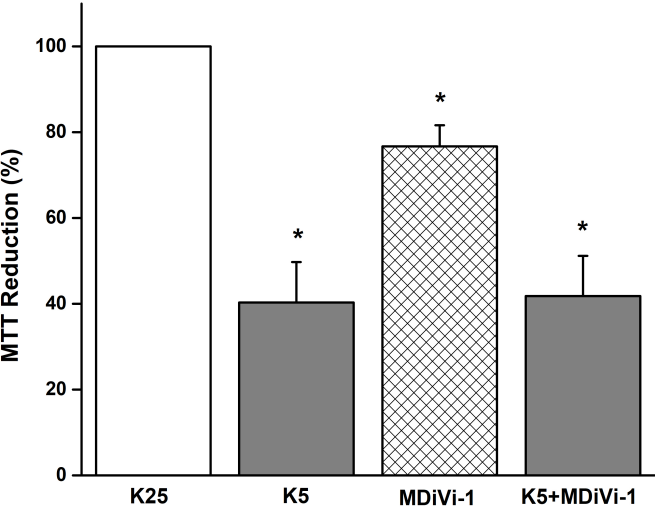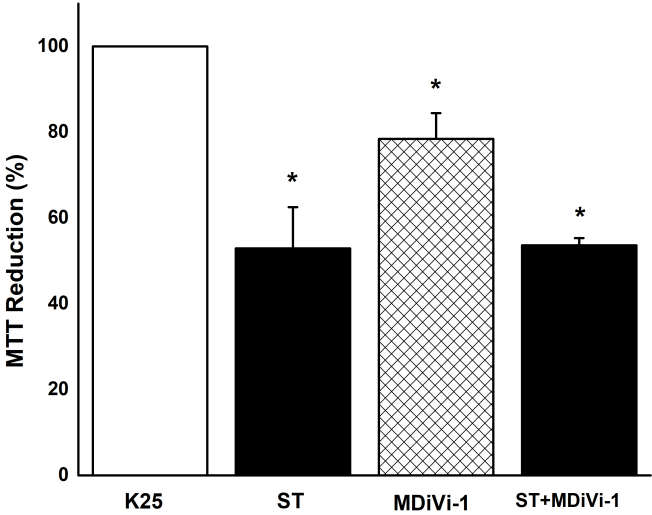

Supplement: Supplementary Materials — Supplementary Figure 1 Effect of MDiVi-1 on cell viability of CGN treated with K5 and ST. Cells were cultured for 7 DIV and treated with staurosporine (0.5 μM) (ST) or transferred to a 5 mM KCl-containing medium (K5) in the presence or absence of the Drp1 inhibitor MDiVi-1 (10 μM); the MDiVi-1 was preincubated 30 min before the corresponding treatment. Cell viability was evaluated as MTT transformation after 24 h. Values are means ± SE of three independent experiments. ∗Significantly different from control (K25) (∗p < 0.05). [file 8832863.f1.pdf]
